# Heart rate variability in male rats

**DOI:** 10.14814/phy2.15827

**Published:** 2023-09-21

**Authors:** Pavol Švorc, Soňa Grešová, Pavol Švorc

**Affiliations:** ^1^ Department of Physiology and Pathophysiology, Faculty of Medicine Ostrava University Ostrava Czech Republic; ^2^ Department of Physiology, Faculty of Medicine University PJ Safarika Kosice Slovak Republic

**Keywords:** heart rate variability, in vivo experiments, rats

## Abstract

The cardiovascular system is primarily controlled by the autonomic nervous system, and any changes in sympathetic or parasympathetic activity also have an impact on myocardial activity. Heart rate variability (HRV) is a readily available metric used to assess heart rate control by the autonomic nervous system. HRV can provide information about neural (parasympathetic, sympathetic, reflex) and humoral (hormones, thermoregulation) control of myocardial activity. Because there are no relevant reference values for HRV parameters in rats in the scientific literature, all experimental results are only interpreted on the basis of changes from currently measured control or baseline HRV values, which are, however, significantly different in individual studies. Considering the significant variability of published HRV data, the present study focused primarily on comparing control or baseline HRV values under different conditions in in vivo experiments involving rats. The aim of the study was therefore to assess whether there are differences in the starting values before the experiment itself.

## 1 INTRODUCTION

There is an existing problem in determining “normal” values of heart rate variability (HRV) parameters in frequency‐domain or time‐domain analyses. Relatively few investigators have used HRV parameters only in terms of changes after a given intervention, derived from baseline values, while not accounting for the fact that the evaluated parameters—or their extent—can be altered already at the beginning of the experiment. Such an approach is understandable given that no standard reference values for HRV in rats have been reported. Chizh (2015) also draws attention to this problem, where he points out the difference in the frequency ranges of various authors, from which spectral components were evaluated. This reflects the considerable variability in baseline or control values at the evaluation of the frequency‐domain or time‐domain HRV parameters themselves. In the present article, we focus on showing differences in control or baseline values of HRV parameters under different conditions in experiments involving rats, which can influence the accuracy of the interpretation of the results and the conclusions drawn.

## 2 HRV IN EXPERIMENTS INVOLVING RATS

The concept that HRV in rodents similarly reflects cardiovascular regulation in humans is supported. Low or missing resting vagus nerve tone in these species, however, can alter the quality and degree of HRV response after a given intervention. Although the origin of variation in the frequency‐domain (Kuwahara et al., 1994) and time‐domain parameters of HRV (Aubert et al., 1999) appears to be analogous to humans, the degree of influence of the parasympathetic division may be greater in rats, as supported by several previous studies (Cerutti et al., 1991; Japundzic et al., 1990).

On the contrary, (Rowan III et al., 2007). queried to what extent changes in HRV can be used as a marker of increased or decreased sensitivity to a given intervention in rodent studies. Perhaps the most appropriate interpretation of most short‐term studies involving rodents is simply that intervention(s) may produce a systemic effect that is mediated either by the nervous system or by the heart or blood vessels, but this is pure speculation and not necessarily supported by concrete evidence. Nevertheless, HRV may still play an important mechanistic role in such research. To better understand the prognostic utility of HRV together with other electrocardiographic (ECG) endpoints, it is necessary to proceed from carefully controlled experiments with known outcomes (Carnevali et al., 2019; Krüger et al., 2000; Sanyal et al., 2002). Therefore, the development of new analytical techniques that connect temporal trends in cardiovascular parameters with pathological conditions is necessary—sometimes even essential—and may provide a basis for future studies using rats with large individual differences in vagus‐mediated HRV.

## 3 HRV: EVALUATION

In the study, we included the values of frequency and time parameters of HRV from studies retrieved from a literature search of the Web of Science database using the keywords “HRV in rat.” HRV parameters that were reported as baseline or control values were summed and means were calculated. In some studies, HRV parameters were reported from the time domain and in others from the frequency domain and, in some cases, HRV was assessed using both methods. Most of the studies described changes in HRV only in graphical form without reporting actual numerical values, or only changes in HRV were noted.

### 3.1 Evaluated groups

Female, juvenile, and old animals were excluded from the study. The age of the rats ranged from 3 to 4 months (i.e., sexually mature male rats). Rat strain and telemetry equipment for HRV measurements were not taken into account. The length of the records from which HRV was evaluated was only described in some methodologies; as such, taking this factor into account was highly problematic. For a clearer comparison of HRV parameters, we created the following groups: *control group*—awake or freely moving animals, HRV was measured telemetrically before the experiment itself; and *sham group*—awake or freely moving animals, in which the animals, after the introduction of telemetric sensing, underwent surgical preparation for the ongoing experiment, but without subsequent targeted experimental intervention.

The most frequently used frequency‐domain parameters (LF power, HF power, LF/HF ratio, LFnu, HFnu and total spectral power (TSP)) and time‐domain parameters (RR interval, SDNN and rMSSD) from selected studies were evaluated and compared.

### 3.2 Statistical analysis

Data are expressed as mean ± standard deviation (SD). Data were analyzed using InStat (GraphPad). The Tukey–Kramer test was used to compare data from the groups, and differences with *p* < 0.05 were considered to be statistically significant. Correlations were calculated as correlation coefficients, in which the coefficient was statistically significant in the range of −0.4 to −1 and from +0.4 to +1.

## 4 HRV: CONTROL (BASELINE) VALUES FROM FREQUENCY‐DOMAIN ANALYSIS

Experimental designs are always based on control or reference values, which fully apply to the activities of the autonomic nervous system. The autonomic nervous system of the rat is very sensitive to various external stimuli and/or those from the internal environment, which can significantly alter the activity of the autonomic nervous system at the beginning of an experiment. Therefore, it is necessary to carefully consider preparation of the animal for the experiment itself and to determine which values to define as the starting point (i.e., baseline) if the desire is to assess not only changes in the autonomic nervous system themselves but also changes in relation to the monitored parameter.

In in vivo experiments involving awake or freely moving animals, baseline values are used from sham groups, when the animals, in addition to the implantation of electrodes for continuous ECG recording, are also subjected to other surgical procedures as preparation for the further course of the experiment, but without subsequent targeted experimental intervention. For example, preparation for coronary or femoral artery occlusion (Aires et al., 2017; De La Fuente et al., 2013; Nobre et al., 2006), catheterization (Beckers et al., 2006; Blanco et al., 2015; Fiorino et al., 2012; Krüger et al., 1997, 2000; Mostarda et al., 2009; Müller‐Ribeiro et al., 2017; Quagliotto et al., 2015; Ramaekers et al., 2002; Ribeiro et al., 2021; Sallam et al., 2016, 2017; Shi et al., 2020; Simoes et al., 2016), ligation (Lima et al., 2018; Maida et al., 2017; Ruiz et al., 2014; Wang et al., 2016), bile duct ligation (Haddadian et al., 2013), cannulation (Beltrán et al., 2020; Da Silva et al., 2002; Neto et al., 2017; Sant'Ana et al., 2011), implantation of stimulators (Dai et al., 2020; Domingos‐Souza et al., 2021), or if they have already been administered substances beforehand (Lo Giudice et al., 2002).

Although the animals used in these experiments undergo a recovery phase (when antibiotics and analgesics are administered), it is not reported anywhere whether the autonomic nervous system modulation over the heart “returns to normal” after recovery from surgical interventions or whether the changes persist from a longer‐term perspective. The overall recovery time after surgery can be a problem. In the methodologies, this time varies from 1 hour to 3–4 weeks and is likely dependent on the severity of the intervention.

In control group, HRV was measured telemetrically before the experiment itself in awake and freely moving male animals (Carll et al., 2012; Choudhary et al., 2018; Couderc et al., 2002; Fazan Jr. et al., 2015; Hazari et al., 2021; Imai et al., 2008; Koizumi et al., 2011; Lamb et al., 2012; Lin et al., 2016; Mamalyga, 2013; Mangin et al., 1998; Pereira‐Junior et al., 2006, 2010; Saalfield & Spear, 2014; Shi et al., 2017; Soler et al., 2018; Towa et al., 2004; Tsai et al., 2020; Yang et al., 2019; Zajączkowski et al., 2014). If we assume that the location of implantation (subcutaneous on the back or intra‐abdominal) and the length of recovery do not play a role, then theoretically we can assume that these values could approach the “missing” reference values, although unfortunately, only for males and at the given time of measurement. On the contrary, also during electrode implantation itself, the animals are anesthetized using different types of anesthetics (pentobarbital, ketamine/xylazine, urethane, and isoflurane), while general anesthesia is known to suppress HRV in human (Matchett & Wood, 2014) and also in animals (Mäenpää et al., 2007).

**FIGURE 1:**
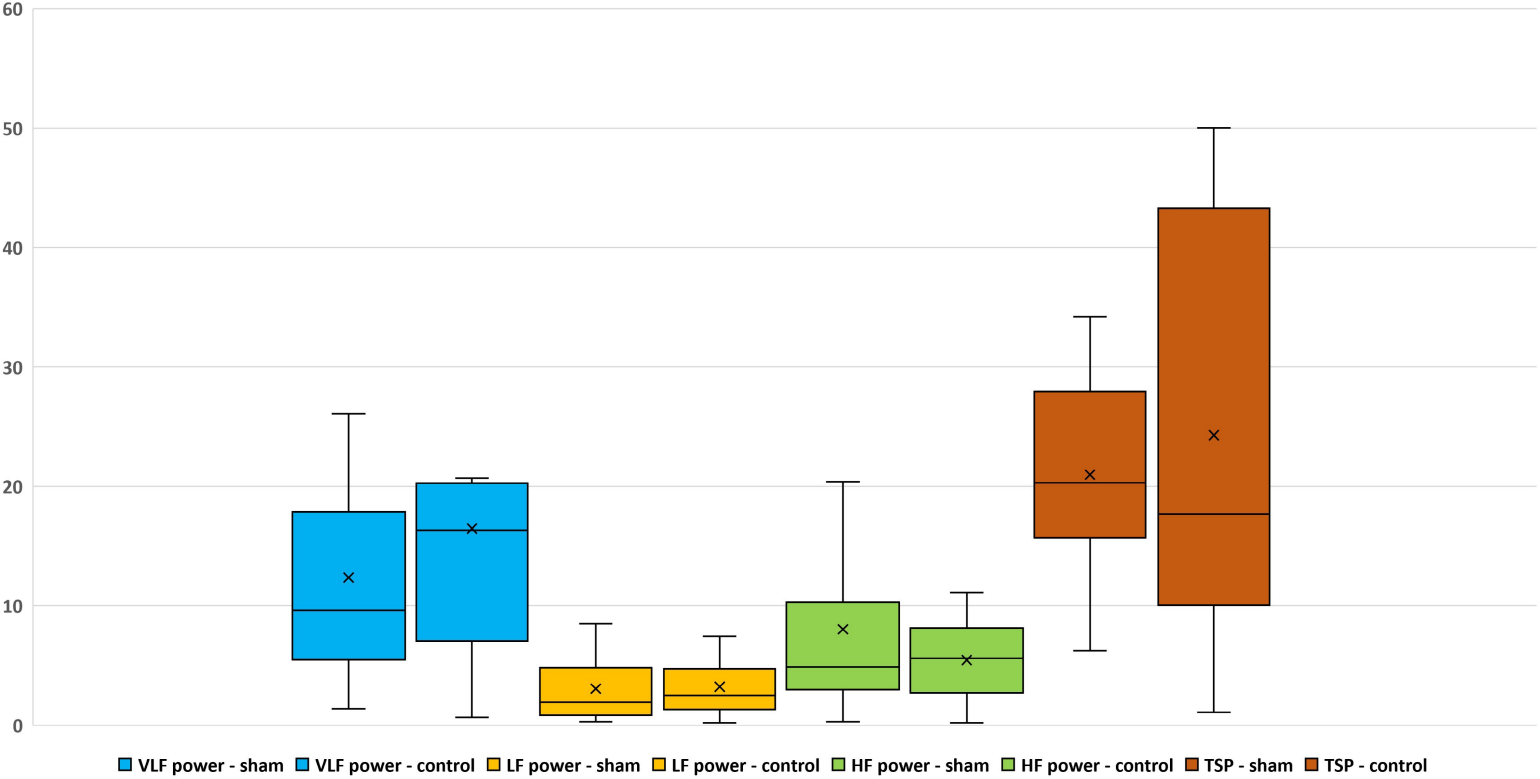
Mean ± SD values of individual frequency HRV parameters from conscious male rats (sham and control). VLF, very low frequency of HRV (VLF‐sham *n* = 6; VLF‐control *n* = 8) LF, low frequency of HRV (LF‐sham n = 34; LF‐control n = 18); HF, high frequency of HRV (HF‐sham *n* = 36; HF‐control *n* = 19); TSP, total spectral power of HRV (TSP‐sham *n* = 12; TPS‐control *n* = 8).

**FIGURE 2:**
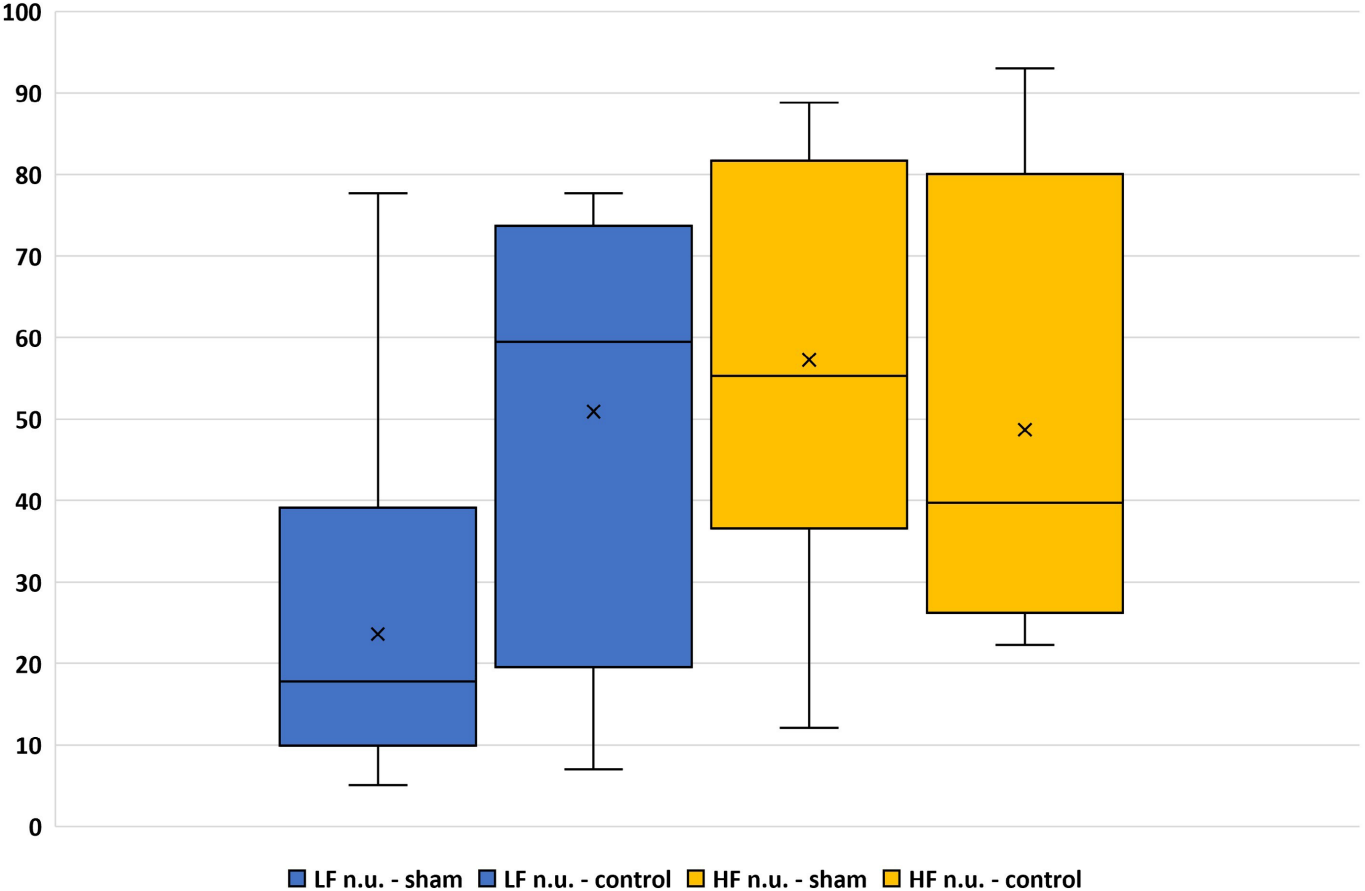
Mean ± SD values of LFnu and HFnu conscious male rats (sham vs. control). LFnu (LFnu‐sham *n* = 26; LFnu‐control *n* = 4) and HFnu (HFnu‐sham *n* = 24; HFnu‐control *n* = 4)—normalized units represent the relative value of the HF and LF components of HRV in relation to TSP (after subtracting VLF power).

To determine whether there are any differences between sham and control groups, we focused only on some evaluated parameters from the frequency‐domain analysis. Differences in baseline values are reported in Table 1 and Figure 1. In the control group, although the HF power (representing the modulation of the parasympathetic) prevails, the LF/HF ratio points to a state in which the sympathetic is significantly dominant. The dominant sympathetic effect is also indicated by the value of LFnu (normalized unit represents the relative value of the LF component of HRV in relation to TSP), which is higher than HFnu (normalized unit represents the relative value of the HF component of HRV in relation to TSP), in contrast to the sham group, in which LFnu is lower than HFnu (Table 1, Figure 2). Based on averaged data, it appears that total HRV (presented by TSP) is reduced in the sham group, although not significantly. Could this mean that the autonomic nervous system in a rat sham group is less sensitive to various manipulations or interventions than the control group? However, this only occurs during the light (i.e., inactive) period of the rat regimen day, when the experiments were performed.

**TABLE 1:** Mean ± SD values of frequency‐domain HRV parameters from conscious male rats.

Group	VLF power (ms^2^)	LF power (ms^2^)	HF power (ms^2^)	LF/HF	LFnu	HFnu	TSP (ms^2^)
Sham	12.4 ± 12.6 (*n* = 6)	3.05 ± 3.06 (*n* = 34)	8.04 ± 9.03 (*n* = 36)	0.75 ± 0.8 (*n* = 33)	23.6 ± 18.8 (*n* = 26)	57.3 ± 23.5 (*n* = 24)	20.98 ± 8.4 (*n* = 12)
Control	16.46 ± 13.34 (*n* = 8)	3.22 ± 2.36 (*n* = 18)	5.45 ± 3.49 (*n* = 19)	1.49 ± 1.46 (*n* = 25) *p* < 0.02	50.9 ± 30.6 (*n* = 4)	48.7 ± 30.7 (*n* = 4)	24.3 ± 17.9 (*n* = 8)

*Note*: Sham—animals which, in addition to the implantation of electrodes for continuous electrocardiographic recording, also underwent other surgical interventions but without targeted experimental intervention; Control—animals that only underwent implantation of electrodes; VLF—is not as well characterized but is believed to reflect circadian inputs to the heart; LF—sympathetic and parasympathetic activities or baroreceptor activity; HF—parasympathetic activity; LF/HF ratio—reflects sympathovagal balance; LFnu, HFnu—normalized units; TSP—presents total HRV; *n* = number of measurements from which the average value was calculated. *p* < 0.02 statistically significant difference between sham and control groups.

Abbreviations: HF, high frequency; LF, low frequency; TSP, total spectral power; VLF, very low frequency.

### 4.1 HRV: control (baseline) values from time‐domain analysis

Similar to investigations that analyzed changes in autonomic nervous system activity using frequency‐domain analysis, other studies have used time‐domain analysis of HRV. As in frequency‐domain studies, baseline values not only in sham groups were used (Abulaiti et al., 2011; De La Fuente et al., 2013; Houshmand et al., 2017; Krüger et al., 1997, 2000; Lima et al., 2018; Müller‐Ribeiro et al., 2017; Sallam et al., 2016, 2017; Silva, Geraldini, et al., 2017; Silva, Silva, et al., 2017; Soler et al., 2018; Wang et al., 2016; Xu et al., 2020), but also from groups in which the values were recorded only before the experimental intervention itself and the animals did not undergo surgical preparation (control) (Barbier et al., 2006; Beckers et al., 2006; Beltrán et al., 2020; Couderc et al., 2002; Farraj et al., 2009; Fazan Jr. et al., 2015; Hashimoto et al., 1999; Hazari et al., 2021; Koresh et al., 2016; Lamb et al., 2012; Lin et al., 2016; Maida et al., 2017; Mangin et al., 1998; Mostarda et al., 2009; Pereira‐Junior et al., 2010; Powell et al., 2021; Ramaekers et al., 2002; Ribeiro et al., 2021; Saalfield & Spear, 2014; Scridon et al., 2012; Shi et al., 2017; Simoes et al., 2016; Zajączkowski et al., 2018).

**FIGURE 3:**
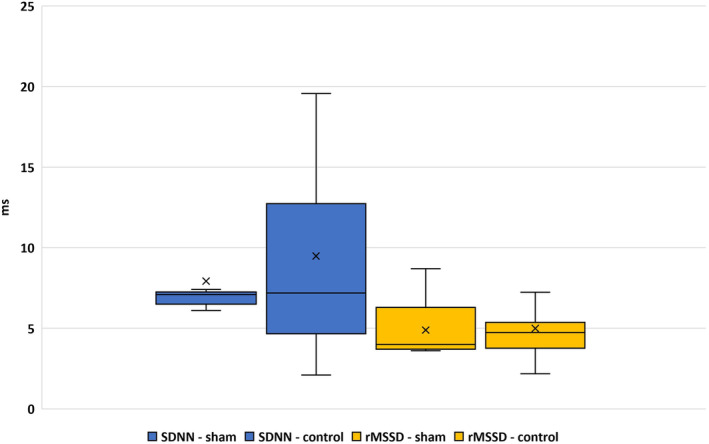
Averaged values of SDNN and rMSSD from conscious male rats (sham and control). SDNN (ms) (SDNN‐sham *n* = 11; SDNN‐control *n* = 25)—a frequently used index of total HRV identical to TSP. rMSSD (ms) (rMSSD‐sham *n* = 7; rMSSD‐control *n* = 23)—higher rMSSD values indicate greater parasympathetic activity.

From data obtained from individual studies involving male rats, significant differences (*p* < 0.001) have been found in the duration of RR intervals between the sham and control groups, while the duration of RR intervals varied depending on parasympathetic activity (rMSSD) in both groups (sham group, *r* = −0.58; control group, *r* = −0.96; Table 2). Based on comparison of the calculated correlation coefficients from both groups, we can only speculate that, in the control group, heart rate is practically and completely dependent on changes in parasympathetic activity, while in the sham group, changes in heart rate can also be the result of the action of other mechanisms. The contribution of the sympathetic nervous system to the duration of the RR intervals from the given data is unclear. Regarding the contribution of total HRV (SDNN) to the duration of RR intervals, a moderate significant dependence was found only in the control group (sham group, *r* = −0.15; control group, *r* = 0.49; Table 2, Figure 3).

**TABLE 2:** Mean (± SD) values of time‐domain HRV parameters from conscious male rats.

Group	Mean RR (ms)	SDNN (ms)	rMSSD (ms)
Sham	157.6 ± 14.5 (*n* = 15)	7.93 ± 3.92 (*n* = 11)	4.89 ± 1.93 (*n* = 7)
Control	170.7 ± 23.2 (*n* = 13) *p* < 0.001	9.48 ± 6.75 (*n* = 25)	4.99 ± 2.24 (*n* = 23)

*Note*: Sham—animals which, in addition to the implantation of electrodes for continuous electrocardiographic recording, also underwent other surgical interventions, but without targeted experimental intervention; Control—animals that only underwent implantation of electrodes; *n* = number of measurements from which the average value was calculated. *p* < 0.001 statistically significant difference between sham and control groups.

## 5 DISCUSSION AND CONCLUSION

Results from each of the cited studies are, of course, excellent. It is obvious in experimental studies that when evaluation of any changes in monitored parameters, it should be based on the control or baseline values. However, the question remains as to why there is so much variability in these values. A follow‐up question would be to what extent the control or baseline values can be—or they are—changed already at the beginning of the experiment itself.

One of the possible problems of great variance HRV may be the nonuniformity in frequency ranges, from which VLF, LF, HF, and TSP are calculated (Table 3). There is also inconsistency in the use of units for HRV frequency parameters, because some investigators have described them in ms^2^, some in ms^2^/Hz, some only in ms, and some not at all. It is assumed that algorithms for the calculation of single frequency and time parameters of HRV should be constant; as such, calculation from different frequency ranges can be one of the causes of variability, because there are enormous differences in the reporting of values, which, in some studies, can differ by orders of magnitude (Chang et al., 2011; Fabiyi‐Edebor, 2020; Malliani et al., 1991; Zhu et al., 2018).

**TABLE 3:** Values of frequency range from individual authors.

Author	VLF (Hz)	LF (Hz)	HF (Hz)	TSP (Hz)
Krüger et al. (2000, 1997)		0.5–0.8	≥0.8	
Mangin et al. (1998)		0.2–1	1–3	
Zajączkowski et al. (2014, 2018) Pereira‐Junior et al. (2006, 2010)		0.2–0.8	0.8–2.5	
Fazan Jr. et al. (2015)	< 0.2	0.2–0.8	0.8–2	
Shi et al. (2017)		0.04–0.6	0.6–2.4	
Mamalyga (2013)	0.02–0,8			
Carll et al. (2012)		0.2–0.8	0.8–2	
Koizumi et al. (2011)		0.3–0.8	0.9–3.3	
Imai et al. (2008) Towa et al. (2004)		0.04–1	1–3	
Shi et al. (2020)	0.01–0.04	0.1–1	1–3	
Ramaekers et al. (2002) Beckers et al. (2006)		0.2–0.7	0.8–2.5	
Couderc et al. (2002)		0.1–0.7	0.7–2	
Beltrán et al. (2020)		0.04–0.6	0.6–2.4	
De La Fuente et al. (2013)	0–0.2	0.2–0.8	0.8–4	
Mostarda et al. (2009) Quagliotto et al. (2015) Blanco et al. (2015) Simoes et al. (2016) Müller‐Ribeiro et al. (2017)	0–0.2	0.2–0.8	0.8–3	
Lo Giudice et al. (2002)	0.03–0.2	0.2–0.6	0.60–3	
Ribeiro et al. (2021) Aires et al. (2017)		0.2–0.8	0.8–3	
Dai et al. (2020)		0.3–0.8	0.8–4	
Wang et al. (2016)		0.1–0.6	0.6–3	
Sallam et al. (2016, 2017)		0.3–0.8	0.8–3	
Abulaiti et al. (2011) Lin et al. (2016)	0.003–0.04	0.04–0.2	0.2–0.4	< 0.4
Lima et al. (2018) Chang et al. (2011)		0.04–0.2	0.2–0.4	
Yang et al. (2019) Tsai et al. (2020)	0.02–0.2	0.2–0.6	0.6–3	0–3
Hazari et al. (2021)		0.2–0.8	0.8–3.5	
Da Silva et al. (2002)		0.2–0.8	0.8–2.5	
Fiorino et al. (2012)		0.2–0.6	0.6–3	
Neto et al. (2017) Maida et al. (2017)	0.01–0.2	0.2–0.8	0.8–3	
Sant'Ana et al. (2011)	0.01–0.2	0.2–0.85	0.8–2.5	
Zhu et al. (2018)	0.003–0.04	0.04–0.2	0.2–0.4	0–0.5

Another problem may be the fact that many methodologies do not describe the interval duration from which individual HRV parameters were evaluated. Different methods of HRV acquisition and evaluation, as well as sex, use of anesthesia during telemetry device implantation, recovery period, and time when the measurement was performed may also be problems (Svorc Jr. et al., 2023).

Analysis of baseline or control values reported in the cited studies clearly reflect wide disparity, and there are no “normal” or standardized reference values for HRV parameters in rats from frequency‐domain or time‐domain analyses of HRV obtained in a specifically defined time of measurement. Therefore, we believe that unequivocal conclusions should not be drawn, especially those regarding the autonomic nervous system and should, therefore, be offered cautiously.

## AUTHOR CONTRIBUTIONS

Pavol Švorc proposed the original draft of the study. Pavol Švorc Jr and Soňa Grešová collected all data and performed data analysis. Pavol Švorc designed the first manuscript. Pavol Švorc and Soňa Grešová drafted the final manuscript. All authors made critical revisions and approved the final version of the manuscript. All authors agree to be accountable for all aspects of the work in ensuring that questions related to the accuracy or integrity of any part of the work are appropriately investigated and resolved. All persons designated as authors qualify for authorship, and all those who qualify for authorship are listed.

## FUNDING INFORMATION

None.

## CONFLICT OF INTEREST STATEMENT

No conflicts of interest, financial or otherwise, are declared by the authors.

## Data Availability

Data were obtained from several scientific articles.
